# 

**Published:** 1864-01

**Authors:** 


Anæsthetics, 33, 34, 228, 287. 332
371, 428, 476, 525
Anthelmintics, ......................33, 218
Amauro is, following Parturition, 37
Army, Health of, ...................... 44
Air in the Veins, ................65, 120
Albuminuria, .. ..................185, 467
Auscultation—Edwards, .......... 193
Atropia ...........................225, 427
American Medical Association, 329
Arsenic in Pemphigus, ............ 332
Asthma, .................... ........ 333
Anatomy and Surgery, Progress
of—Fergusson .................. 352
Aggregate Labor of Mankind, ... 426
Aloes in Treatment of Wounds, 456

BOOK NOTICES,
Military Medicine—Woodward, 45
On the Teeth—Robertson, ....... 47
Mental Hygiene—Ray .......... 48
Hospital Gangrene—Goldsmith, 92
Materia Medica, Synopsis of
Lectures on—Carson, .......... 95
Proceedings of American
Pharmaceutical Association, 186
Physiology—Dalton, ............ 187
Medical Formulary—Ellis, ..... 188
American Medical Association,
Transactions of, ............... 234
Medical Diagnosis—DaCosta, 281
A Treatise on Pharmacy—
Parrish, ..... ................ ..... 283
Obstetrics—Hodge, ............. 375
Venereal Diseases—Bumstead, 379
Dose and Symptom Book—
Wythe, ...... . .................. 380
Diseases of Women—Scanzoni, 380
Military Medical and Surgical
Essays, ......... .................. 565
Surgical Diagnosi—McLeod, ... 566
Medical Dictionary—Thomas, 568
Army Surgeon's Manual, ....... 569
Slade on Diphtheria, ............ 569

Bone, Growth of, ...................... 331
Bromide of Potassium, .............. 364
Births, Proportion to Population, 475

Correspondence—Dr. Cicatrix, .... 62
" Editorial, .. ..... 136
" ...........267. 268, 269
Consumption Curers of N. Y., .... 164
Case for Argument ....... ......... 206
Creosote and Arsenic in Skin
Diseases ........................ 276
Cerebro-Spinal Meningitis—Allen, 241
" " " McVey, 310
" " " Eaton, 312
Code of Medical Ethics, ........... 315
Congestion, Observations on—
J. A. A., .......................... 337
Caustics, ............................... 448
Calabar Bean, ............... ........ 463
Communicated—Jones, ..... ...... 534

Dislocation of Humerus, ............ 37

" " Hip Joint, ......... 409

Drowning, Its Treatment, ......... 63

Dysentery, ............................. 168

Diphtheria—Millard, ............... 201

" Metcalfe, .........272 412

Disease. Observations on—J.A.A. 383

" Treatment of by Controlling Circulation, .......... 401

Diphtheria, Spurious.... ...... ... 470

Duties and Responsibilities of the Physician—Hoag, ....... 539

Eye, Clinical Lectures on—Holmes,
20, 55, 114, 152, 195, 258, 290,
344, 393, 443, 509, 545
Empyema, Treatment of,—Slayter, 149
Eczema, ................................ 174
"Excito Secretory," ............... 266
Ear, Foreign Bodies in, ......... 473
Electricity, New Use of, ........... 525
Ergot of Wheat, Effects of, ....... 558

Fermentation as a cause of Disease, ... 30
Fœtus in Utero, Nervous and Vascular Connection of, ... 141

Gall-Stones, ... 170

Gutta Percha Tree, ... 223

Gonorrhœa, ... 363

Grafting Animals, ... 427

Hospital Gangrene,—Cleveland, 1
Hemorrhage, post-partum—Brooks, 49
Hippocrates. Tomb of, ... 475
Homœopathic Association of Ill., 278

Iodine, Uses of, ... 29
Indigenous Drugs, ... 183
Intestine, Perforation of, ... 199
Inflammation of Fauces, ... 207
Iodide of Lime, ... 334
Introductory Address—Miller, ... 479

Ligature of the Sub-Clavian, ... 89
" of the Common Iliac —Brainard, ... 97
" Femoral Artery, ... 476
La Pommerais Case, ... 462
Lungs, Weight of, ... 472
Labor, Complicated Case of, ... 514

Morton Fund Association, ... 42
Malaria—Munson, ... 102
Medical Societies, Proceedings of,
Montgomery County, Ind., ... 157
Aurora, Ills., ... 158
Illinois State, ... 180, 230
Galen Medical, Stark Co., Ind, 522
Medical Matters in Vicksburg, ... 181
McMunn's Elixir, ... 229
Medical Graduates of 1864, ... 280
Mortality in Chicago, ... 286
Metropolypus—Trimble, ... 303
McPherson Hospital, Vicksburg, 372
Medical Journals, Suspension of, 520

Neuralgia over Vertebral Spine, 524

OBITUARY NOTICES,
C. H. Cleveland, M. D., ... 96
John C. Dalton, M. D., ... 96
R. L. Smith, Student, ... 42
Shubal York, M. D., ... 189
O. L. Leonard, M. D., ... 190
Franklin Bache, M. D., ... 191
L. P. Cheney, M. D., ... 284
John Rodman Cox, M. D., ... 284
Robert P. Thomas, M. D., ... 286
Professor Ribes, ... 286
James Miller, M. D., ... 476
Jonathan Knight, M. D., ... 522
Orthopedic Surgery—Prince, ... 431, 495, 527
Opisthotonos Cured by Ice, ... 557

Pharmacopœia, Squibb's Report, 25
Pneumonia, ... 39
Physicians in Chicago, ... 43
Physiology and Pathology—Bennett, ... 160
Pulmonary Congestion in Children, 184
Pessaries—Eaton, ... 391
Pancreatic Juice, ... 426
Puerperal Convulsions ... 459

Resolutions of Medical Class, ... 42
Rush Medical College, ... 89, 519
Rambles in Military Hospitals—Lackey, ... 145, 300
Rheumatic Fever—Wade, ... 175, 208

Scarlet Fever, ... 43
Sanitary Commission, ... 82, 421
Surgeon-General U. S. A., ... 85, 419
Scrotum, Tumor of,—Baxter, ... 309
Senna, ... 333
Surgeon of the Alabama, ... 369
Strychnia, Antidotes for, ... 474
Syphilis, Constitutional, ... 549

To our Patrons, ... 38
Trousseau, Resignation of, ... 43
Trichiniasis, ... 213
Tape Worm, Male Fern in Treatment of, ... 218
" " Pepo in Treatment of —Ingals, ... 263
Tobacco, Physiological Effects of, 556
Tetanus, ... 559

Uterus. Inversion of,—Eaton. ... 60
Urethra, Stricture of,—Slayter, 99
Ulcers About the Nails, ... 173
" Chronic, ... 286
" ... 343, 400, 564
Uterus, Foreign Body in,—Meachem, ... 350
" Prolapsus of,—Slayter, ... 389
Uterine Pain, Hypodermic Treatment of, ... 462
Uterine Hydatids, ... 562

Vaccination, ... 41, 44
Varicose Veins, ... 22, 267, 425

### EDITORIAL.

*To Our Patrons.*—The present number commences the 21st Vol. of the CHICAGO MEDICAL JOURNAL, counting the aggregate of the different series. The custom that has formerly obtained, when its proprietorship has been changed, of repeating the numbers of the volumes, but making them of a new series, has been productive of inconvenience, and we have therefore thought best, as making the reference to any particular article more easy, to number this volume in accordance with our whole issue, and we hope in future that this practice may not be changed. Few publications of any kind in the North-west have had a continued existence so long as this, and in the whole nation, but a small number of medical ones. It was established when Chicago was but an inconsiderable town, and the garden of the North-west but little inhabited. It has witnessed the transformation of this small town into a substantial city of more than 150,000 inhabitants, and with a business greatly disproportionate to the number of its people. It has seen the then unoccupied virgin prairies, fructified by the touch of industry, suddenly teeming with vast wealth, and inhabited by a numerous people, unsurpassed for intelligence, enterprise, courage, and fidelity to their country. In the profession, it has witnessed changes of a no less salutary nature, and of equal magnitude, with the material resources of both city and country. The JOURNAL has been a medium of communication between members of the profession embraced in a wide extent of country, and to the very marked progress made during this period, in scientific attainment, in medical knowledge, and practical skill, it may justly claim to have contributed its share of influence. We predict that the two decades to come will bring progress no less marvelous than the two that are past, have shown—and with those who shall carry on this work, the JOURNAL will be a faithful co-laborer, as in times gone by. We are under great obligation to the profession for the steady support they have always so generously accorded us. Our list of subscribers has constantly increased, while remittances of pecuniary aid have been more and more prompt and universal.

We hope to make the current volume an interesting and useful one. We are promised a series of articles from Prof. Brainard on ununited fractures. We shall publish a series of Dr. Holmes' clinical lectures before the class of Rush Medical College, on the diseases of the eye, the first of which will be found in the present number. Other able writers have promised us contributions, and we solicit communications from all who will favor us by transmitting interesting articles for our pages. With this number we send bills for all dues up to the present volume—and though the bill for this is withheld, our patrons will not forget that our terms are *payment in advance*. Though most of our subscribers have paid with praiseworthy promptitude, yet a few are considerably in arrears. To all, this number will be sent, but those who have unpaid bills of much amount, and which shall remain unpaid on the issue of the next number, will have their names struck from the list, and the bills will be given to an agent for collection.

